# Genome-Wide Association Integrating a Transcriptomic Meta-Analysis Suggests That Genes Related to Fat Deposition and Muscle Development Are Closely Associated with Growth in Huaxi Cattle

**DOI:** 10.3390/vetsci12020109

**Published:** 2025-02-02

**Authors:** Cheng-Li Liu, Tao Ren, Peng-Cheng Ruan, Yong-Fu Huang, Simone Ceccobelli, De-Jun Huang, Lu-Pei Zhang, Guang-Xin E

**Affiliations:** 1 College of Animal Science and Technology, Southwest University, Chongqing 400715, China; lcl222333@outlook.com (C.-L.L.); mangran1145@163.com (T.R.); 15368890603@163.com (P.-C.R.); h67738337@swu.edu.cn (Y.-F.H.); 2 State Key Laboratory of Animal Biotech Breeding, Institute of Animal Sciences, Chinese Academy of Agricultural Sciences, Beijing 100193, China; 3 Department of Agricultural, Food and Environmental Sciences, Università Politecnica delle Marche, 60131 Ancona, Italy; s.ceccobelli@staff.univpm.it; 4 Chongqing Academy of Animal Science, Chongqing 402460, China; xkyhdj@163.com; 5 Institute of Animal Sciences, Chinese Academy of Agricultural Sciences, Beijing 100006, China

**Keywords:** GWAS, RNA-seq, muscle development, fat deposition, Huaxi cattle

## Abstract

Growth traits are among the most important economic phenotypes targeted in the genetic improvement of beef cattle. To understand the genetic basis of growth traits in Huaxi cattle, we performed a genome-wide association study (GWAS) on body weight, eye muscle area, and back fat thickness across five developmental stages in a population of 202 Huaxi cattle. Additionally, publicly available RNA-seq data from the longissimus dorsi muscle of both young and adult cattle were analyzed to identify key genes and genetic markers associated with growth in Huaxi cattle. In total, 7.19 million high-quality variant loci (SNPs and INDELs) were identified across all samples. In the GWAS, the three multilocus models (FarmCPU, MLMM, and BLINK) outperformed the conventional single-locus models (CMLM, GLM, and MLM). Consequently, GWAS analysis was conducted using multilocus models, which identified 99 variant loci significantly associated with growth traits and annotated a total of 83 candidate genes (CDGs). Additionally, 23 of the 83 CDGs overlapped with significantly differentially expressed genes identified from public RNA-seq datasets of longissimus dorsi muscle between young and adult cattle. Furthermore, gene functional enrichment (KEGG and GO) analyses revealed that over 30% of the pathways and GO terms were associated with muscle development and fat deposition, crucial factors for beef production. Specifically, key genes identified included *MGLL*, *SGMS1*, *SNX29* and *AKAP6*, which are implicated in lipid metabolism, adipogenesis, and muscle growth. In summary, this study provides new insights into the genetic mechanisms underlying growth traits in Huaxi cattle and presents promising markers for future breeding improvements.

## Simple Summary

Beef is an important source of protein for humans, but the yield of meat is directly influenced by growth traits. Therefore, breeding new beef cattle breeds with fast growth rates is particularly important in the beef cattle industry. In this study, we used genome-wide association analysis combined with RNA-seq data to screen many key genes that may be involved in the growth and development of Huaxi cattle. The findings provide significant evidence for the genetic mechanisms underlying growth traits and will assist in the marker-assisted breeding of fast-growing varieties.

## 1. Introduction

With the rapid development of the global economy and the continuous improvement of human living standards, the demand for beef has reached an unprecedented level [1]. The economic growth traits of beef cattle have become breeding improvement programs [2]. Genome-wide association studies (GWASs) have identified several candidate genes (CDGs) related to key economic traits, such as body weight, eye muscle area (EMA), and backfat thickness (BFT), of various beef cattle breeds. For example, *PLIN3* has been shown to be associated with the body weight of Simmental beef cattle and is an important regulator of adipogenesis and triglyceride storage [3,4]. Genes such as *IGF1* influence body weight across developmental stages in beef cattle [5]. Additionally, *IGF1* can activate the expression of myogenic regulatory factors, thereby promoting the proliferation and differentiation of bovine myoblasts [6]. Many genes associated with BFT and EMA have been confirmed to be involved in muscle development and fat deposition. For instance, *CTNNA1*, *AADAT*, and *CACNA2D1* are associated with BFT in cattle [7]. *CTNNA1* acts as an inhibitor of myogenesis, which leads to reduced muscle development [8]. In addition, *CACNA2D1* is involved in mammalian fat deposition [9]. From a physiological perspective, fat deposition and muscle development are the primary contributors to animal weight and meat quality [10]. Some genes (e.g., *AKAP6*, *IGF1*, and *MSTN*) related to cattle growth and development have been confirmed to play roles in fat deposition and muscle development [5,11].

Huaxi cattle (HXC) is a newly developed specialized beef breed in China, established over approximately 40 years of systematic crossbreeding and genetic improvement, with lineage contributions from Simmental, Charolais, Sanhe, and Mongolian cattle [12]. This breed is characterized by rapid growth, strong adaptability, and high reproductive performance. It plays an important role in the sustainable development of beef cattle industry in China [13]. However, limited research exists on candidate markers and key genes associated with economically important traits in HXC, such as growth. 

In this study, a GWAS was conducted on seven growth traits of HXC, identifying 99 significant variants and 83 CDGs. By integrating published RNA-seq data, 23 significantly differentially expressed candidate genes (DE-CDGs) were identified in the longissimus dorsi muscle between calves and adult cattle. These findings provide valuable scientific insights for the breeding and genetic improvement of HXC.

## 2. Materials and Methods

The experimental procedures of this study were authorized by the Ethics Committee of Southwest University (Approval Number: IACUC-20240710-07). A total of 202 HXC individuals were obtained from the Jinxia Cattle Breeding Farm in Chongqing (E108°42’, N30°54’, altitude of 793 m). The farm also provided data on various growth performance indicators collected at different developmental stages, including birth weight (BW), 6-month weight (6-MW), 12-month weight (12-MW), 18-month weight (18-MW), 24-month weight (24-MW), 18-month BFT, and 18-month EMA. BFT and EMA were measured by ultrasound (Eastern bell, China) between the 12th and 13th ribs of 18-month-old cattle. A total of 5 mL of tail vein blood was collected from each individual, and genomic DNA was extracted using the TIANamp Genomic DNA Kit according to the manufacturer’s instructions (TIANGEN, China). Sequencing libraries were generated by the VAHTS Universal DNA Library Prep Kit for the MGI platform (Vazyme, China) and sequenced for ~15 Gb per individual on the DNBSEQ-T7 (Beijing Genomics Institute, China). 

The raw sequencing data were processed and filtered using FASTP v.0.20.0 software, and a total of 26.958 billion high-quality clean reads (HQRs) were obtained (the average Q30 was 92.8%). The HQRs were mapped to the *Bos taurus* reference genome (ARS-UCD2.0) by BWA-MEM v0.7.17-r1188 (https://github.com/lh3/bwa, accessed on 15 September 2024). Single-nucleotide polymorphism (SNP) and insertion–deletion (INDEL) variants were identified by GATK v4.2.4.1 (https://github.com/broadinstitute/gatk accessed on 20 September 2024) and further filtered by VCFtools v0.1.16 excluding variants with missing values > 10% and minor allele frequencies < 5%. Finally, 7,195,880 high-quality SNPs and INDELs were obtained for further analysis.

Principal component analysis (PCA) was conducted using the Genome Association and Prediction Integrated Tools (GAPIT) software (https://github.com/jiabowang/GAPIT accessed on 29 September 2024). GWAS results were displayed using GAPIT v.3 software with six algorithm models: general linear model (GLM), mixed linear model (MLM), compressed MLM (CMLM), multiple-loci mixed model (MLMM), fixed and random model circulating probability unification (FarmCPU), and Bayesian-information and linkage-disequilibrium iteratively nested keyway (BLINK) [14]. To address the issue of multiple comparisons and establish phenotype-specific significance thresholds, a permutation-based approach was employed. For each phenotype, the phenotype labels were permuted 100 times while preserving the original genotype data. GWAS was performed on each of the 100 permuted datasets using GAPIT (an MLM model), and the minimum *p*-value (MP) among all SNPs was recorded for each permutation. This process generated a null distribution of the MPs, which inherently reflects the multiple testing burden for the given phenotype [15]. The empirical significance threshold for each phenotype was calculated as the average of these 100 minimum *p*-values. This permutation-based approach inherently accounts for multiple comparisons and provides a robust, data-driven significance threshold tailored to the statistical characteristics of each phenotype, including its variance, distribution, and noise levels [14]. Unlike traditional methods such as Bonferroni correction, this approach avoids excessive conservatism, which may otherwise reduce the ability to detect true associations. The significance thresholds calculated for the seven phenotypes were as follows: 6.08 × 10⁻⁷ (BW), 2.31 × 10⁻⁷ (6-MW), 5.21 × 10⁻⁷ (12-MW), 9.42 × 10⁻⁷ (18-MW), 9.85 × 10⁻⁷ (2-MW), 6.61 × 10⁻⁷ (BFT), and 9.11 × 10⁻⁷ (EMA), respectively. 

Additionally, publicly available RNA-sequencing (RNA-seq) data from the longissimus dorsi muscle of cattle, including young cattle (320–403 days postnatal, n = 10) and adult cattle (900–930 days postnatal, n = 10), were downloaded from the NCBI database (Table S1). The raw data were processed using FASTP (v0.2), and ribosomal RNA was removed using BOWTIE2 (v2.3.5.1) to obtain high-quality clean reads. These clean reads were aligned to the reference genome (*Bos taurus*, ARS-UCD2.0) using HISAT2 (v2.1.0). Transcript assembly and gene expression levels were visualized with StringTie (v2.1.1). Gene expression quantification and differential expression analysis were performed using DESeq2 (v1.26.0). Significance thresholds were set at |log2(fold change) | > 1 and FDR < 0.05, where the false discovery rate (FDR) was controlled using the Benjamini–Hochberg method to correct for multiple testing.

The variants were annotated using ANNOVAR (http://www.openbioinformatics.org/annovar/, accessed on 14 October 2024), which integrates annotation databases compatible with the Generic Feature Format v.3 (GFF3). The annotation process included mapping the variants to various genomic regions, such as 3′ untranslated regions (3′UTR), 5′ untranslated regions (5′UTR), introns, and exons. Additionally, genes located within 100 kb upstream and downstream of intergenic SNPs were evaluated. The functional enrichment of CDGs was conducted using Gene Ontology (GO) and Kyoto Encyclopedia of Genes and Genomes (KEGG) databases via KOBAS (http://bioinfo.org/kobas, Bos taurus, accessed on 15 November 2024). Statistical significance for enrichment was determined using the hypergeometric test, and pathways with a corrected *p*-value < 0.05 were considered significant.

## 3. Results

The PCA results revealed that the first three components (PC1, PC2, and PC3) accounted for 2.58%, 1.59%, and 1.57% of the total genetic variation, respectively (Figure S1). These findings indicate the absence of significant structural differentiation within the population (Figure S2). A statistical analysis of the seven economic traits (BW, 6-MW, 12-MW, 18-MW, 24-MW, BFT, and EMA) in HXC showed that they followed a Gaussian distribution (Figure S3, Table S2).

The GWAS was performed using six different statistical models to minimize the risk of false positives and false negatives in identifying candidate markers. The QQ plots for the single-locus models demonstrated that the observed *p*-values closely matched the expected *p*-values across the five body weight traits, with no significant loci identified. Notably, in the analyses of the two carcass traits using the three single-locus models, most points on the QQ plots were located below the diagonal line, indicating that the observed *p*-values were lower than expected (Figure S4). This suggests a high occurrence of both false positives and false negatives in the single-locus models (MLM, GLM, and CMLM). In contrast, the three multilocus models (FarmCPU, BLINK, and MLMM) showed better alignment with the expected diagonal line (Figure S4). This pattern indicates that the multilocus models establish a genuine association while effectively managing false positives and negatives. Consequently, we annotated and analyzed the significant loci identified by the three multilocus models.

**Figure 1:**
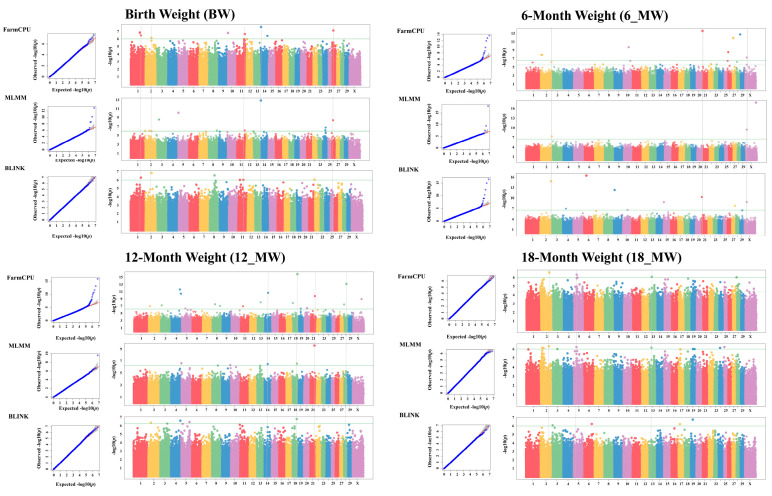
QQ plots and Manhattan plots for BW, 6-MW, 12-MW, and 18-MW. The green lines represent the significant threshold. QQ plots are displayed as scatter plots of observed and expected –log10 (*p*-values). Different colored dots in the Manhattan map indicate variation sites on different chromosomes.

**Figure 2:**
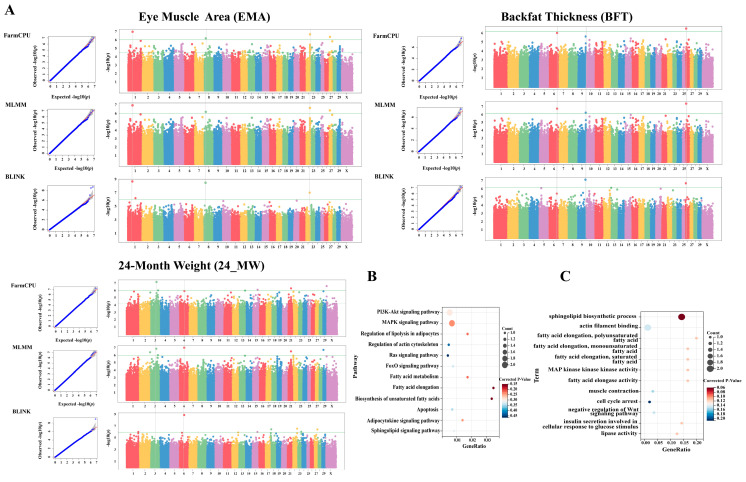
GWAS of EMA, BFT and 24-MW, and enrichment analysis of candidate genes related to all growth traits. (**A**), QQ plots and Manhattan plots for EMA, BFT, and 24-MW. The green lines represent the significant threshold. QQ plots are displayed as scatter plots of observed and expected –log10 (*p*-values). Different colored dots in the Manhattan map indicate variation sites on different chromosomes.(**B**) Signaling pathways related to muscle development and fat deposition in the KEGG functional enrichment analysis of the CDGs. (**C**) Terms related to muscle development and fat deposition in the GO functional enrichment analysis of the CDGs.

The GWAS using the multilocus models identified 99 variant loci significantly associated with the seven phenotypes (Figure 1 and Figure 2A; Table S3). Notably, five loci, including snp_18_58135746, snp_26_8880660, and snp_8_24264659, were consistently identified by all three GWAS models. Additionally, 15 loci, such as snp_1_117590967, snp_2_133554064, and snp_5_39067941, were detected by two models simultaneously. Interestingly, most loci associated with the two carcass traits were identified by multiple models concurrently.

In total, the candidate loci were annotated with 83 candidate genes (CDGs) and 29 non-coding RNAs (Table S3). KEGG enrichment analysis revealed that 27 CDGs (e.g., SGMS1, GYPC, and MGLL) were involved in 96 signaling pathways, including morphine addiction, the PI3K-Akt signaling pathway, and fatty acid metabolism. Among these pathways, 13% (e.g., fatty acid elongation, biosynthesis of unsaturated fatty acids, and regulation of lipolysis in adipocytes) were directly related to muscle development and fat deposition (Figure 2B; Table S4). GO enrichment analysis mapped 60 CDGs to 367 GO terms, encompassing processes such as protein homodimerization activity, lipid metabolism, and sphingolipid biosynthetic process (Table S5). Notably, over 5% of these GO terms were directly associated with muscle development and fat deposition (Figure 2C). 

**Figure 3:**
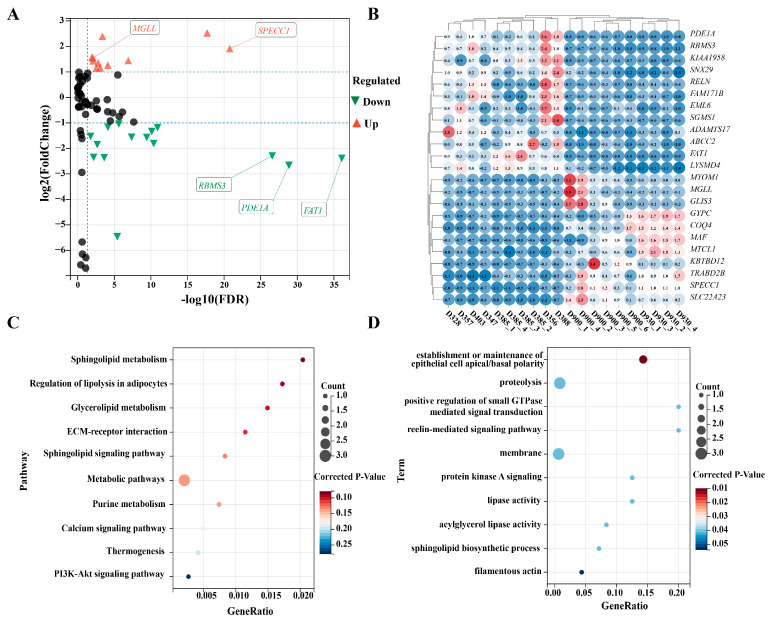
Functional analysis of DEGs between the young and adult cattle. (**A**) Volcano plot of 83 CDGs; red represents upregulated expression and green represents downregulated expression; black represents no significant. (**B**) Clustering heat map of 23 DE-CDGs; red represents upregulated expression and blue represents downregulated expression. (**C**) Signaling pathways related to muscle development and fat deposition in the KEGG functional enrichment analysis of the DE-CDGs. (**D**) Terms related to muscle development and fat deposition in the GO functional enrichment analysis of the DE-CDGs.

To further explore the potential roles of CDGs in muscle development and fat deposition in cattle, we analyzed their expression levels in the longissimus dorsi muscle of young and adult cattle using publicly available RNA-seq data. A total of 7166 genes were identified as significantly differentially expressed between the two developmental stages (Table S6). Notably, 23 of the 83 CDGs were significantly differentially expressed (Figure 3A), with 11 upregulated and 16 downregulated (Figure 3B; Table S7), highlighting their potential involvement in bovine muscle development and fat deposition. Functional enrichment analysis revealed that eight DE-CDGs were mapped to twenty-six KEGG signaling pathways, including the PI3K-Akt, metabolic, and calcium signaling pathways. Over 30% of these pathways (e.g., regulation of lipolysis in adipocytes, PI3K-Akt, and ECM-receptor interaction) were directly related to muscle development and fat deposition (Figure 3C; Table S8). GO enrichment analysis showed that 21 DE-CDGs were significantly enriched in 128 GO terms, such as the establishment or maintenance of epithelial cell apical/basal polarity, proteolysis, and positive regulation of small GTPase-mediated signal transduction. Many of these terms, including proteolysis, positive regulation of small GTPase-mediated signal transduction, and lipase activity, were directly or indirectly associated with muscle development and fat deposition (Figure 3D; Table S9).

## 4. Discussion

Genome-wide association studies (GWASs) require suitable statistical models to accurately uncover genotype–phenotype associations. Single-locus models, such as the mixed linear model (MLM) and general linear model (GLM), are popular due to their simplicity and computational efficiency [16]. However, these models analyze SNPs individually, limiting their ability to capture interactions, epistasis, and polygenic effects, which are essential for traits with complex genetic architectures [17]. They are also prone to false-positive and false-negative rates, particularly in populations with strong linkage disequilibrium (LD) or population structure [18]. In contrast, multilocus models simultaneously analyze multiple SNPs, improving power and precision by accounting for polygenic effects and interactions [14,19]. Multi-point models like FarmCPU, BLINK, and MLMM have been proven effective for complex quantitative traits [20]. These models effectively control for confounding factors and provide a holistic understanding of genetic architecture, making them particularly advantageous for livestock traits, including growth, carcass composition, and fertility [21]. Evidence from studies in beef cattle and yak populations shows that multilocus models consistently identify key quantitative trait loci (QTL) and yield better reproducibility across populations and environments [21,22]. This study’s dual approach, using both single-locus and multilocus models, allows for a direct comparison of their effectiveness, highlighting the limitations of single-locus models and the advantages of multilocus methods in identifying the genetic basis of economically important traits.

Several DE-CDGs identified in this study have also been implicated in previous research on livestock traits. For instance, *MGLL* and *RELN* have been shown to correlate significantly with fat deposition and carcass traits in pigs [23,24], while the *GYPC* gene is linked to carcass traits in multiple cattle breeds [25]. Furthermore, mutations in the *SNX29* gene have been associated with body size, weight, and carcass traits in pigs [26], goats [27], and cattle [28]. However, the precise mechanism by which *SNX29* influences animal growth and development remains unclear. 

Previous studies have demonstrated that the period from birth to 12 months of age represents a critical phase of rapid muscle growth in cattle, followed by a gradual decline in growth rate as they age [29,30]. In this study, 23 genes associated with HXC growth were identified through GWAS, and transcriptomic analysis revealed their significant differential expression in the longissimus dorsi muscle at two distinct developmental stages. These genes were significantly enriched in pathways related to muscle development and fat deposition, including the regulation of lipolysis in adipocytes, the PI3K-Akt signaling pathway, and ECM–receptor interaction [31]. These findings highlight the potential roles of these DE-CDGs as key regulators of growth and development in cattle. Furthermore, many of these genes have been linked to muscle and skeletal development. For instance, *AKAP6* has been identified as a regulator of myoblast differentiation, myotube formation, and muscle regeneration [32]. It promotes *MYOG* expression through *MEF2A*, which, in turn, enhances *AKAP6* expression, forming a positive feedback loop [32]. In this study, a significant decline in *AKAP6* expression was observed in adult cattle (log2 [fc] = −0.28, FDR < 0.05). Similarly, *Adamts17* is involved in bone formation through the regulation of the BMP–Smad1/5/8 pathway [33]. And variations in *Adamts17* have been linked to short stature [34]. As a paralog of *SPECC1*, *SPECC1L* directly binds to *MYPT1*, influencing the distribution of the *MYPT1/PP1β* complex between microtubules and filamentous actin networks [35]. The knockdown of *SPECC1L* in oocytes has been shown to cause abnormal spindle morphology, chromosomal misalignment, and reduced developmental capacity, polar body extrusion rates, and blastocyst formation [36].

Although the rate of muscle growth in cattle decreases with age, the deposition of intramuscular fat increases in most cattle breeds [37,38]. In this study, many DE-CDGs associated with fat deposition were significantly downregulated in the longissimus dorsi muscle of adult cattle (FDR < 0.05). Specifically, *SGMS1* plays a key role in sphingomyelin and lipid metabolism [39] and promotes the osteogenic differentiation of mesenchymal stem cells through the Cer/PP2A/Akt signaling pathway [40]. Interestingly, *SGMS1* deficiency has been linked to adipose tissue atrophy, reduced lipoprotein lipase activity, elevated plasma triglyceride levels, impaired fatty acid uptake, and malnutrition [41,42]. In this study, *SGMS1* expression was significantly upregulated in the longissimus dorsi muscle of young cattle but significantly downregulated in adults. These findings suggest that *SGMS1* is essential for maintaining adipose tissue homeostasis and supporting body growth. Moreover, *RELN* signaling, which is influenced by dietary factors, has been implicated in central nervous system lipid metabolism. Alterations in central CF-RELN levels impact food intake and body weight by modulating synaptic signaling in ARH-POMC neurons [43]. Furthermore, *RELN* receptors, *ApoER2* and *VLDLR*, are also involved in lipid metabolism [43]. Additionally, *RELN* receptors, *ApoER2* and *VLDLR*, play crucial roles in lipid metabolism. The absence of *ApoER2* in bone-marrow-derived macrophages accelerates obesity and diabetes onset, while its deficiency in other tissues leads to hyperglycemia and inflammation due to defective insulin secretion [44]. Similarly, *VLDLR* expression is regulated by *PPARγ*, contributing to lipid uptake and lipogenesis [45]. Finally, transcriptome analyses have demonstrated that *RELN* is associated with fat deposition in sheep, further supporting its role in lipid metabolism and adipogenesis [46]. 

This study also demonstrates that DE-CDGs associated with fat deposition exhibit high expression levels in the longissimus dorsi muscle of adult cattle. Among them, *MYOD* enhances *MAF* expression during myogenesis by binding to the E-box promoter region of *MAF* [47]. *MAF* expression increases during the differentiation of mesenchymal stem cells into muscle cells but is suppressed by *PPARγ* inhibition during the adipogenic differentiation of mesenchymal fibroblasts [47] but decreases under *PPARγ* inhibition during the differentiation of mesenchymal fibroblasts into adipocytes [47]. In addition, high *MAF* expression in small intestinal cells promotes differentiation, enhancing lipid digestion and absorption [48]. Conversely, the absence of *MAF* can impair nutrient and bile acid absorption in the intestine [48]. Another critical metabolic enzyme, *MGLL*, regulates fat deposition and lipid biosynthesis by converting triglycerides into free fatty acids [49]. It plays a dual role in lipid metabolism, facilitating fat breakdown and fatty acid release in adipocytes [50]. *MGLL* knockout in mice reduced weight gain, plasma triglyceride levels, and liver triglyceride accumulation while improving insulin sensitivity and glucose tolerance [51]. Conversely, *MGLL* overexpression in the mouse small intestine significantly increased weight gain, body fat accumulation, and triglyceride levels in both the liver and plasma [52]. Interestingly, *MGLL* overexpression in forebrain neurons reduced endogenous cannabinoid levels, leading to decreased weight gain [53]. 

This study identified several loci, such as snp_22_51003978 and snp_1_117590967, which were consistently associated with growth traits in Huaxi cattle across multiple models. Related genes have also been shown to be involved in muscle and fat development in animals. Interestingly, *ARIH2*, *EIF2A*, and *ELOVL4* did not exhibit significant expression differences in the longissimus dorsi muscle between young and adult cattle, suggesting that their functional roles might be prominent in other tissues, such as the liver or adipose tissue, or during distinct developmental stages. For example, *ARIH2* acts as a key regulator of myogenic differentiation by influencing proteasome-mediated protein degradation and stabilizing critical myogenic transcription factors [51]. Studies have shown that *PDHB* and *FoxP1* modulate ARIH2-induced myoblast differentiation by interacting with upstream signaling pathways [51]. High *ARIH2* expression can restore induced myogenic defects [54]. Genome-wide selective sweep analysis has linked ARIH2 mutations to variations in meat quality across cattle breeds [11]. *ALK* also regulates myoblast differentiation and myotube size [55], and its knockdown can reverse the inhibitory effect of BMP9 on myoblast differentiation [56]. 

eIF2α, a eukaryotic translation initiation factor, is pivotal for protein synthesis and plays a critical role in regulating muscle protein synthesis during growth and development [57,58]. The phosphorylation of *eIF2α* controls C/EBPα/β and *PPARγ* expression, thereby influencing liver fat deposition and glucose metabolism [59]. The activation of *PPARγ* promotes ceramide synthesis and upregulates key lipid metabolism genes, including *CERS3* and *ELOVL4*. As part of the fatty acid elongation system, *ELOVL4* catalyzes the rate-limiting initial step in fatty acid elongation [60,61]. It may also contribute to intramuscular fat deposition and marbling formation in cattle [62]. Mutations in *ELOVL4* mislocalize the protein in the endoplasmic reticulum, disrupting fatty acid elongation [63]. In addition, an exon 2 mutation (snp_8_65120621) in *ERp44* was significantly associated with body weight (BW) in this study (*P* = 2.635 × 10⁻⁷). The covalent bond between *ERp44* and Cys39 is essential for adiponectin maturation and release. *ERp44* dose-dependently suppresses adiponectin complex secretion [64,65]. Conversely, *ERp44* deficiency leads to reduced blood glucose levels, lipid synthesis disorders, and impaired myocardial development in mammals [66]. Further studies are required to elucidate the regulatory mechanisms of these CDGs in cattle growth and development.

## 5. Conclusions

This study demonstrated that multilocus models outperformed single-locus models, identifying 99 variant loci associated with growth and carcass traits, including body weight and backfat thickness in HXC. Among the 83 CDGs identified through GWAS, 23 exhibited significant differential expression in the longissimus dorsi muscle between young and adult cattle, underlining their involvement in growth and development (e.g., *RELN*, *SGMS1*, and *AKAP6*). These findings contribute to a deeper understanding of HXC growth genetics and provide valuable markers for breeding improvement.

## Data Availability

The data of whole genome sequencing have been deposited in the NCBI database with the accession number PRJNA1164013. All data of RNA- seq were cited and downloaded from the NCBI database.

## Supplementary Materials

The following supporting information can be downloaded at https://www.mdpi.com/article/10.3390/vetsci12020109/s1. Figure S1. Principal component analysis of 202 HXC; Figure S2. Heat map of the relatedness of 202 HXC; Figure S3. Histogram of phenotypic value frequency distribution of 7 growth traits; Figure S4. Manhattan plot of GWAS analysis of growth traits in HXC using three single-site models; Table S1. Sample information for RNA-seq; Table S2. Descriptive statistics of phenotypes; Table S3. List of the significant SNPs associated with trait in the HXC; Table S4. KEGG functional enrichment analysis of CDGs; Table S5. GO functional enrichment analysis of CDGs; Table S6. Differentially expressed genes in the longissimus dorsi muscle from both young and adult cattle; Table S7. DE-CDGs in the longissimus dorsi muscle of young and adult cattle; Table S8. KEGG functional enrichment analysis of DE-CDGs; Table S9. GO functional enrichment analysis of DE-CDGs.

## Abbreviations

**Abbreviations**	**Full Names**
GWAS	Genome-wide association analysis
CDGs	Candidate genes
HXC	Huaxi cattle
SNPs	Single nucleotide polymorphism
INDELs	Insertion and deletion
DE-CDGs	significantly differentially expressed candidate genes
GO	Gene Ontology
KEGG	Kyoto Encyclopedia of Genes and Genomes
EMA	Eye muscle area
BFT	Backfat thickness
BW	Birth weight
6-MW	6-month weight
12-MW	12-month weight
18-MW	18-month weight
24-MW	24-month weight
MP	The minimum *p*-value
GLM	General linear model
MLM	Mixed linear model
CMLM	Compressed MLM
MLMM	Multiple loci mixed model
FarmCPU	Fixed and random model circulating probability unification
BLINK	Bayesian-information and linkage-disequilibrium iteratively nested keyway
HQRs	High-quality clean reads
PCA	Principal component analysis
3′ UTR	3′ untranslated regions
5′ UTR	5′ untranslated regions
