# Supplementary material for: Genome-Wide Association Integrating a Transcriptomic Meta-Analysis Suggests That Genes Related to Fat Deposition and Muscle Development Are Closely Associated with Growth in Huaxi Cattle
Source: Vet Sci. 2025 Feb 2;12(2):109. doi: 10.3390/vetsci12020109 (PMC11860805; doi:10.3390/vetsci12020109)
Supplement: Supplementary file 1 [file vetsci-12-00109-s001.zip › Figure S3 Histogram of phenotypic value frequency distribution of 7 body conformation.pdf]

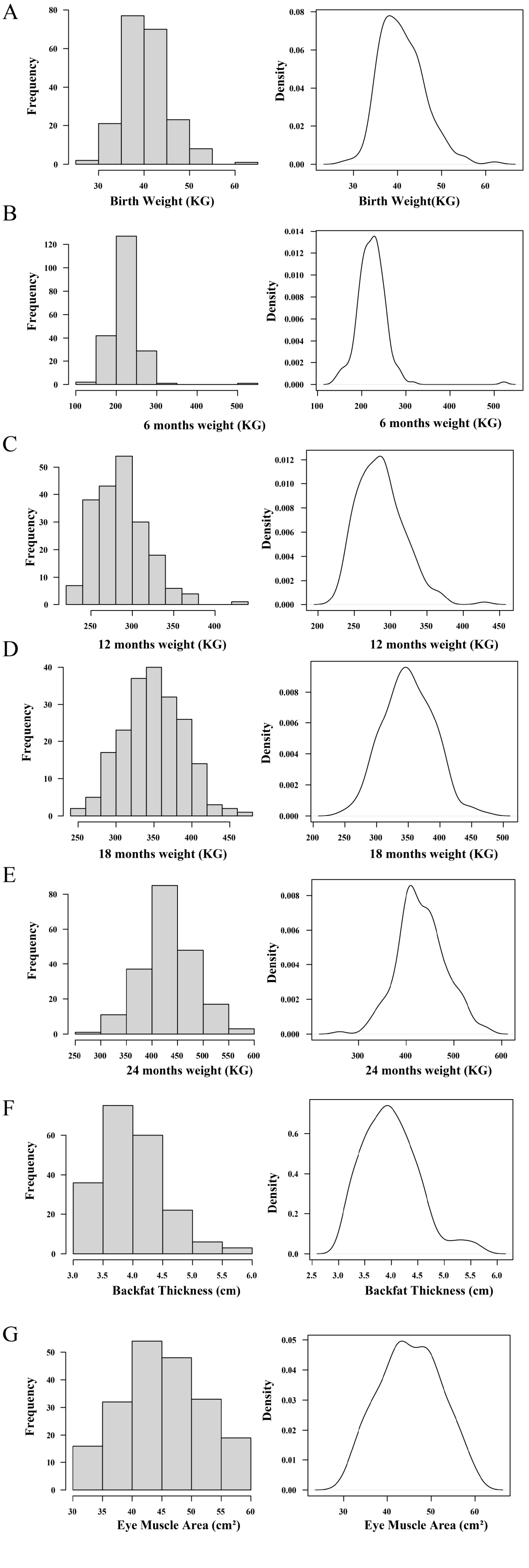
A
Frequency
Birth Weight (KG)
Density
Birth Weight(KG)
B
Frequency
6 months weight (KG)
Density
6 months weight (KG)
C
Frequency
12 months weight (KG)
Density
12 months weight (KG)
D
Frequency
18 months weight (KG)
Density
18 months weight (KG)
E
Frequency
24 months weight (KG)
Density
24 months weight (KG)
F
Frequency
Backfat Thickness (cm)
Density
Backfat Thickness (cm)
G
Frequency
Eye Muscle Area (cm²)
Density
Eye Muscle Area (cm²)

Figure S3 Phenotypic value distribution of 7 growth trait. (A) Birth Weight (BW), (B) 6 months weight (6-MW), (C) 12 months weight (12-MW), (D) 18 months weight (18-MW), (E) 24 months weight (24-MW), (F) Backfat Thickness (BFT), (G) Eye Muscle Area (EMA).
