# Supplementary material for: Genome-Wide Association Integrating a Transcriptomic Meta-Analysis Suggests That Genes Related to Fat Deposition and Muscle Development Are Closely Associated with Growth in Huaxi Cattle
Source: Vet Sci. 2025 Feb 2;12(2):109. doi: 10.3390/vetsci12020109 (PMC11860805; doi:10.3390/vetsci12020109)
Supplement: Supplementary file 1 [file vetsci-12-00109-s001.zip › Figure S4. GWAS analysis of growth traits in HXC using three single-locus models..pdf]

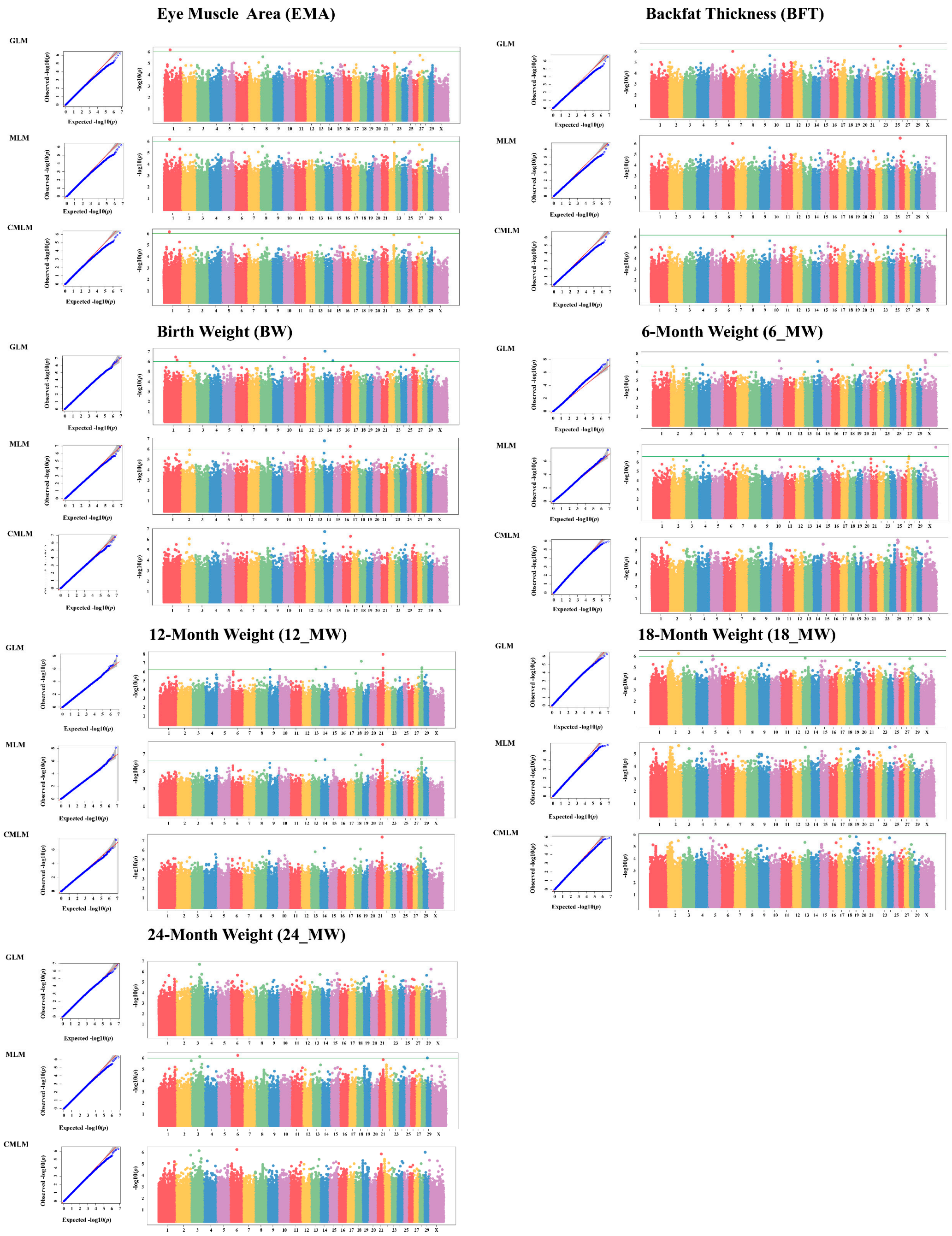

Figure S4. GWAS analysis of growth traits in HXC using three single-locus models. QQ plots and Manhattan plots for EMA, BFT, BW, 6-MW, 12-MW, 18-MW, and 24-MW, respectively. The green lines represent the significant threshold. QQ plots are displayed as scatter plots of observed and expected –log10(p-values).
