# Supplementary figures and images for: Genome-Wide Association Integrating a Transcriptomic Meta-Analysis Suggests That Genes Related to Fat Deposition and Muscle Development Are Closely Associated with Growth in Huaxi Cattle
Source: Vet Sci. 2025 Feb 2;12(2):109. doi: 10.3390/vetsci12020109 (PMC11860805; doi:10.3390/vetsci12020109)

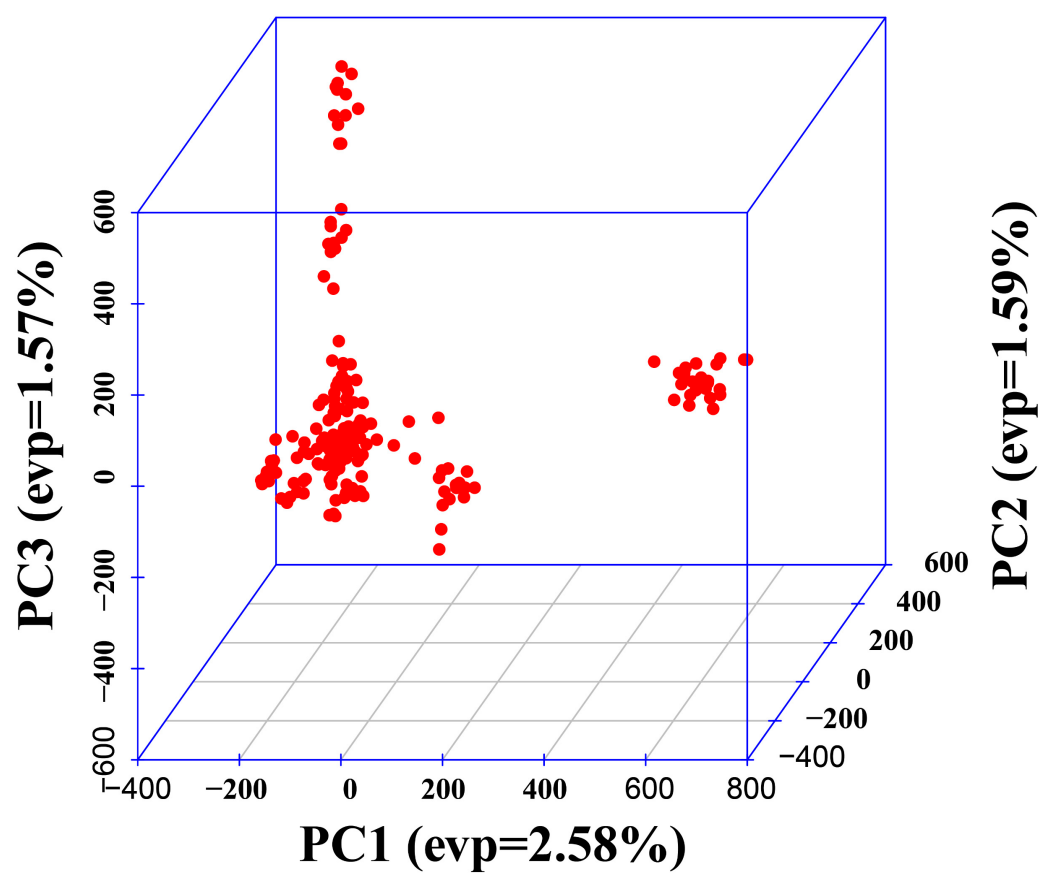

Figure S1. Principal component analysis of 202 Huaxi cattle.

Supplement: Supplementary file 1 [file vetsci-12-00109-s001.zip › Figure S1 Principal component analysis of 202 HXC.pdf]

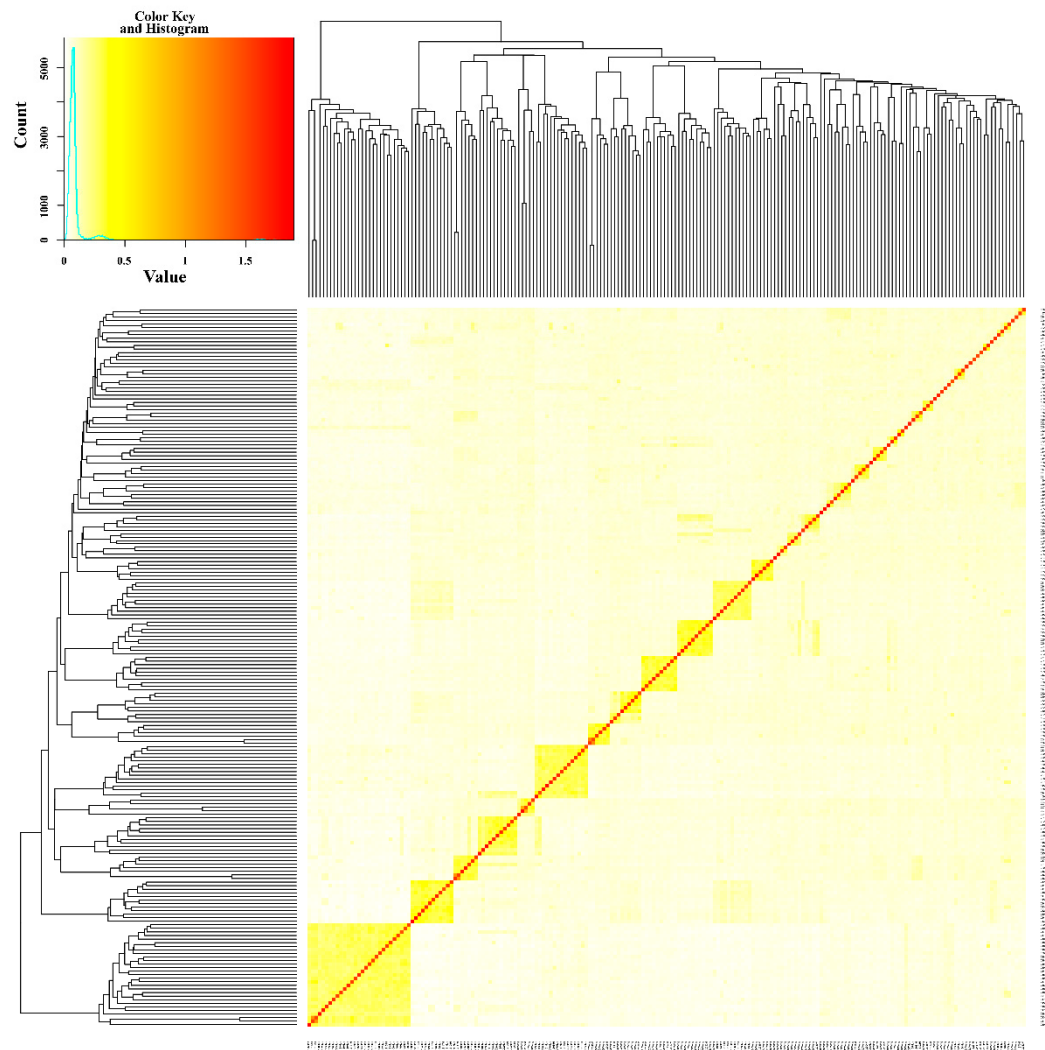

Figure S2 Heat map of the relatedness of 202 Huaxi cattle

Supplement: Supplementary file 1 [file vetsci-12-00109-s001.zip › Figure S2 Heat map of the relatedness of 202 HXC.pdf]
